# Etherization in Midwifery Practice

**Published:** 1847-10

**Authors:** 


*Etherization in Midwifery Practice.*—In the *Gazette Médicale de Strasbourg*, there is a case given by M. Stoltz, where *turning* was deemed expedient, and before anything to that effect was done, the patient was put under the influence of the vapour of ether until insensibility to external impressions was produced. The case presenting nothing peculiar in itself, it will suffice to say that the ether neither diminished the resistance of the uterus to the introduction of the hand, nor facilitated the version or extraction of the fœtus. No accident followed to the mother from its employment however. The child had been dead on her admission into hospital.—*Ibid.*

